# Acute pediatric appendicitis in black and white: clinical disparities, impact and future recommendations

**DOI:** 10.3389/fped.2024.1453927

**Published:** 2024-10-30

**Authors:** Aimen Waqar Khan, Marrium Sultan Dar, Rayyan Nabi, Ahmad Ali, Muhammad Abdullah Humayun, Eman Riaz

**Affiliations:** ^1^ Department of Accident and Emergency, Jinnah Postgraduate Medical Center, Karachi, Pakistan; ^2^ Department of Internal Medicine, Medical ICU, Jinnah Postgraduate Medical Center, Karachi, Pakistan; ^3^ Department of Internal Medicine, Islamic International Medical College, Riphah International University, Karachi, Pakistan; ^4^ King Edward Medical University, Lahore, Pakistan; ^5^ Department of Pediatrics, Rawalpindi Medical University, Rawalpindi, Pakistan; ^6^ Department of Internal Medicine, Ayub Medical College, Abbottabad, Pakistan

**Keywords:** pediatric, appendicitis, disparities (health racial), appendectomy, inequity

## Abstract

Racial and ethnic disparities have long been studied in the delivery of healthcare. One such avenue is acute pediatric appendicitis, which continues to be an area of significant and continual research. Because of its routine clinical presentation and standardized management, acute pediatric appendicitis serves as an appropriate proxy for studying discrepancies in healthcare. Our review therefore aims to comprehensively highlight the various dimensions of its clinical management subject to disparities, their collective clinical impact, and future recommendations to mitigate it.

## 1 Introduction

The most frequent surgical emergency in pediatrics is appendicitis, which accounts for 10% of all pediatric cases presenting to the emergency department (ED) with abdominal pain (1). On the other hand, the initial missed diagnosis (MD) rate ranges from 28% to 57% for children under the age of 12 to nearly 100% for those under the age of 2 (2). The most significant complication associated with delay in diagnosis is appendiceal perforation (3) which may thereafter result in abdominal abscess formation, bowel resection, multiple abdominal surgeries, sepsis, and intensive care unit hospitalization (4, 5). These complications in turn translate into longer hospital stays and increased healthcare costs.

These statistics are alarming as, in spite of its generally routine clinical presentation, diagnostic delays in the management of acute appendicitis still exist in children. While these delays may be attributed to a variety of factors like the absence of a pathognomonic picture and difficulties in eliciting a history and proper physical exam (2, 6), there exist significant racial and ethnic disparities in its routine management that put minority children at a heightened risk of complications (7, 8). Our review therefore aims to collate the various disparities in the clinical management of acute pediatric appendicitis, underline their collective clinical impact, and explore recommendations to mitigate it.

## 2 Defining race, ethnicity, disparities, and inequity

Before delving into a discussion on its impact, it is critical to comprehend the meanings of race and ethnicity and how they may translate into disparities and inequity.

Race can be conceptualized as a social and cultural framework that divides people into groups based on perceived or self-identified physical attributes. On its own, it is without any genetic or physiological basis (9) and the singular confines of race grossly undervalue the substantial variability within phenotypic groupings (10, 11). Ethnicity on the other hand groups individuals on cultural and traditional grounds, being more of a socio-political construct. Boundaries of ethnicity are fluid and subject to change (12). The difference between racial and ethnic groups is important to understand. Examples of racial groups include Asian American, African or Black American, American Indian or Native American, and Caucasian or White American. On the other hand, ethnicity refers to cultural identity. People of Hispanic ethnicity can belong to different racial groups, such as White, Black, or Asian. Additionally, individuals of sub-Saharan African ethnicity are almost exclusively Black racially, while Pacific Islander ethnicity often correlates with the Asian racial group (12). For standardized reporting in economic and epidemiologic studies, the US Office of Management and Budget (OMB) categorizes race and ethnicity into commonly used designations (13, 14). The Institute of Medicine Report defines disparities as variations in healthcare quality resulting from recognized or unrecognized bias or discrimination (9). On the other hand, inequity refers to “Differences in health and well-being outcomes that are avoidable, unfair, and unjust. Health inequities are affected by social, economic, and environmental conditions.” (15).

Having said this, we shall now take a look at how racial and ethnic disparities translate into healthcare inequities.

## 3 Materials and methods

**Figure 1:**
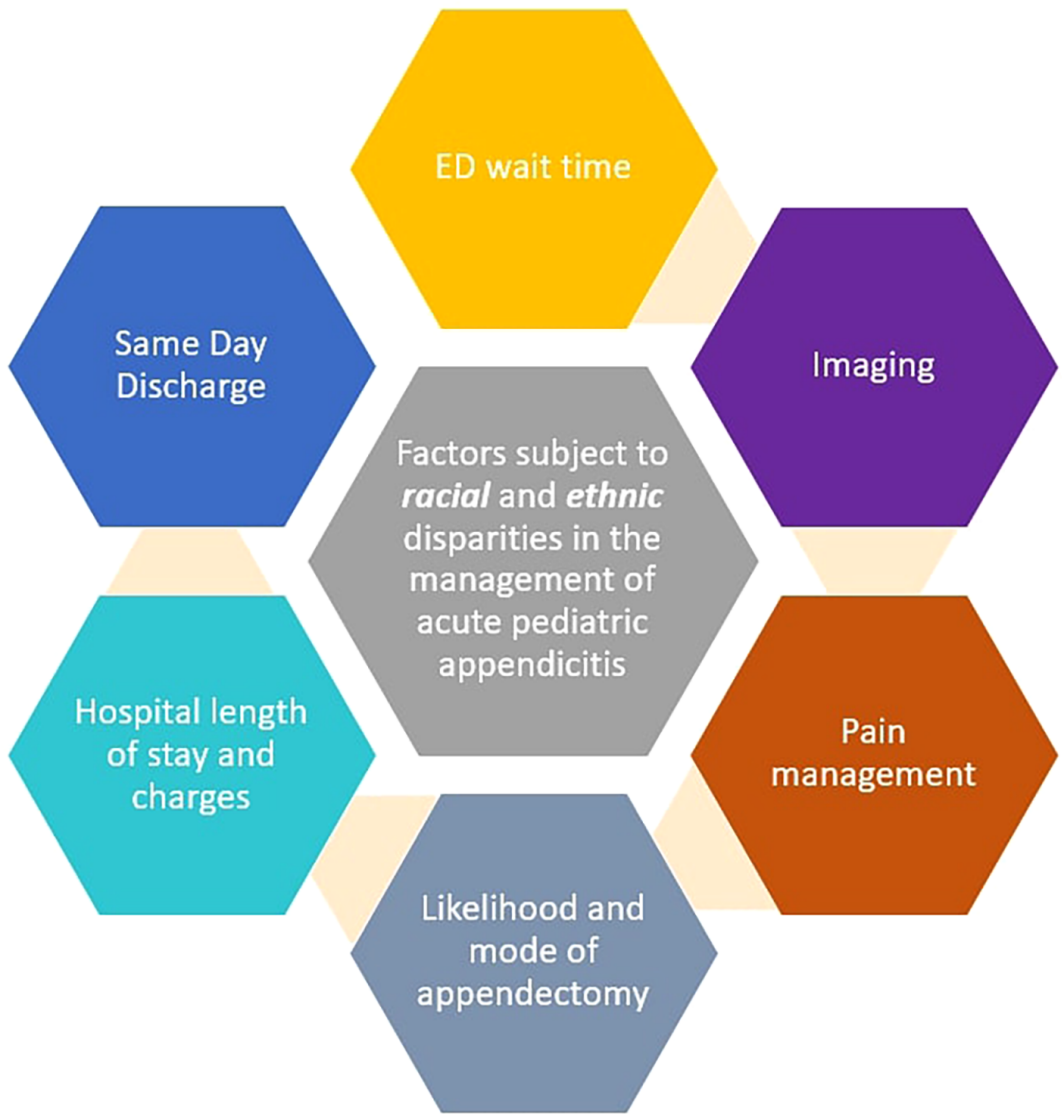
Racial and ethnic disparities noted in the management of acute pediatric appendicitis.

Our review article entailed a comprehensive literature review involving PUBMED/MEDLINE and Google Scholar databases from inception till June 2024. The keywords used were: [(“racial groups”) OR (“racial disparities”)] AND [(“ethnic”) OR (“ethnic disparities”)] AND (“health disparities”) AND [(pediatric) OR (child)] AND [(“acute appendicitis”) OR (“appendicitis”)]. We selected all articles published across the globe that explored racial and ethnic discrepancies in the management of acute pediatric appendicitis. Data from both rural and urban systems were included. The majority of the included studies were conducted in the United States (US). Articles were only excluded if they were published in a language other than English. We then summarized all the key areas with noted clinical disparities, their collective impact, and explored recommendations to mitigate them (Figure 1).

## 4 Factors subject to racial and ethnic disparities

### 4.1 Wait times

The amount of time spent in the ED between arrival and the initial medical evaluation by a doctor or advanced practice provider is known as the “wait time” and is a key indicator of the quality of care provided (16). Timeliness in care can not only significantly alter the prognostic outcome of acutely distressing conditions by preventing organ dysfunction and mortality (17), but also reduce hospital admission rates (18) and improve ED visit satisfaction (19). Multiple studies have differently explored the racial and ethnic disparities in the quality of pediatric emergency department (PED) care (20, 21), including PED wait times across broad diagnoses using large national datasets (22). These wait times vary among various racial and ethnic groups in pediatric appendicitis. According to research, Non-Hispanic White (NHW) children had a 33% shorter PED wait time than non-Hispanic Black (NHB), Hispanic, and other racial group's children (*p* < 0.001) (16). While this striking disparity in wait time may be accounted for by variables like differences in severity, crowding, and demographics, non-White patients were still found to experience 12% greater wait times after controlling for confounders than White patients. A study conducted in US found that there were notable variations in the triage status reported by 93 million children under the age of 15, with just 14.6% of NHW patients falling into the >2-hour immediacy category, while 18.8% of NHB patients and 20.0% of Hispanic White patients were in this range (22). Park et al.'s study carried out in the US also affirms the above findings by demonstrating 10.4% greater wait times for Hispanic children than non-Hispanic children and the difference was statistically significant (*p* < 0.05) (23).

### 4.2 Imaging

While acute appendicitis is frequently the primary cause of acute abdominal pain, clinicians often encounter challenges in making this diagnosis (24).To improve diagnostic accuracy, a number of scoring methods have been established, keeping in mind the challenge of striking a balance between the rate of perforations and the rate of negative appendectomies (25, 26). However, when scoring systems are equivocal or in atypical cases, imaging in the form of ultrasound (US) and computed tomography (CT) is indicated to counter the rate of false-negative diagnoses, reduce morbidity from perforation, and lower hospital expenses (27). According to the most recent American College of Radiology guidelines, patients with suspected acute appendicitis, in whom right lower quadrant US is equivocal or non-diagnostic, the next usually appropriate study would be MRI or CT of the abdomen and pelvis with or without contrast (28). Not infrequently, there exist disparities in terms of race and ethnicity in the decision to undergo imaging, and even when that decision is made, there is variation in the choice of imaging modality for clinching the diagnosis. For instance, according to a study conducted in the US, compared to White patients, Black patients had significantly (*p* < 0.05) lower rates of advanced imaging (e.g., US or CT) in over 20,000 pediatric visits for abdominal pain (29).

### 4.3 Pain management

Analgesia is essential for treating acute appendicitis since it is the most common cause of surgical abdominal pain (30). Pain control may be achieved by a variety of modalities including drugs like opioids or non-opioid analgesics (31) [e.g., acetaminophen and nonsteroidal anti-inflammatory drugs (NSAIDs)] and nerve blocks which are used infrequently (32). Contrary to older beliefs, the administration of analgesia is strongly endorsed in favor of improved patient cooperation and comfort, with large, randomized studies now dispelling any reservations about patient safety, masking of symptoms, and diagnostic accuracy (33, 34). Indeed, pain control is advocated to the extent that withholding analgesia unless contraindicated is considered unacceptable (35). However, between adult and pediatric patients presenting with presumed appendicitis, the rates of analgesia administration in pediatric patients are grossly underwhelming (36). Goyal et al. utilized the Stanford Comparative Pain Scale according to which pain data from 2009 to 2010 were reclassified into four categories: no pain, mild pain, moderate pain, and severe pain. The indication for analgesia administration was determined based on these pain thresholds, ensuring appropriate pain management for patients. According to studies conducted in the US, even amongst the pediatric cohort, racial disparities concerning analgesia administration and specifically opioid administration exist with Black patients being half as likely to receive analgesia as White patients [adjusted odds ratio = 0.2 (95% CI, 0.06–0.8)] (37, 38). Moreover, a higher clinician threshold for pain has also been noted for Black patients with appendicitis. For example, among patients with moderate intensity pain, Black persons were only 15.7% likely to receive analgesia vs. 58.5% of White patients [adjusted odds ratio = 0.1 (95% CI, 0.02–0.8)]. Similar trends were also noted with severe pain, with Black persons being only 24.5% likely to receive opioid analgesia compared to a 58.3% likelihood in White patients [adjusted odds ratio = 0.2 (95% CI, 0.06–0.9)] (37). It must also be noted that while racial disparities were striking, no ethnic disparities (Hispanic vs. Non-Hispanic) were observed about opioid administration. A plausible explanation is the observation that because Hispanic children tend to be more likely to present with a perforated appendix than uncomplicated appendicitis, they were not found to experience ethnic discrepancies on that front (39).

### 4.4 Likelihood of surgery and mode of appendectomy

An overall clinical picture and certain risk factors play a crucial role in assessing whether a patient requires surgical or conservative management; however, appendectomy has emerged as the preferred treatment for acute appendicitis in all age groups, following fluid resuscitation, analgesia, and intravenous antibiotics (40)**.** With benefits such as a shorter postoperative ileus, lesser analgesic requirements, shorter hospital stays, and lower rates of wound infections and subsequent adhesive bowel blockage, laparoscopic appendectomy is considered superior to open appendectomy as the surgical option (41)**.** However, it has been established that laparoscopy is not an option equitably available to all. Race and ethnicity have been identified as the key patient-level factors associated with surgical disparities among the population across broader disease categories (42)**.** Several studies utilizing data from U.S. registries have been conducted (43–45). Lack of timely surgical care, combined with other factors, has been linked to an increased incidence of perforation in Black Hispanic children having appendicitis compared to White children (24% and 19%, respectively) (46). Moreover, African American and Hispanic children are less likely to undergo procedures like laparoscopic appendectomies, which are typically a more appropriate course of treatment. According to a study by A. Oyetunji et al., only 6.4% of Black children and 35.5% of Hispanic children underwent laparoscopic appendectomy, compared to 50.5% of Caucasian children and the difference was statistically significant (*p* = 0.005) (43, 44)**.**

### 4.5 Hospital length of stay (LOS) and hospital charges

Hospital length of stay (LOS), which is linked to higher hospital expenses and complications, has long been used as a metric to evaluate the quality of care (45, 47). For children with uncomplicated appendectomies, the mean post-operative LOS is postulated to be between 0 and 2 days (48). However, growing evidence underscores significant racial and ethnic disparities in hospital LOS, as evidenced by studies that utilized data from US registries (49, 50). Research by Harrington et al. demonstrated that Black children had hospital stays that were 12.4% longer than White children (*p* < 0.001), with Hispanic children having stays that were 4.5% longer (*p* < 0.001) (49). Similarly, another study demonstrated an increased use of surgical drains, open surgical procedures, fever following operation, post-operative imaging, prolonged LOS, and an increase in the number of ED visits following surgery in Hispanic children as opposed to non-Hispanic White children (*p* < 0.0001) (50). While these discrepancies in LOS may be attributed to the increased prevalence of appendiceal perforation and complicated appendicitis noted at the time of presentation in Black and Hispanic children (51, 52), still it is important to realize that inequities in access to healthcare services and socioeconomic constraints underlie the root of the issue (53). Black and Hispanic children thus have lengthier hospital stays and ensuing hospital costs.

### 4.6 Same-day discharge following appendectomy

Same-day discharge (SDD) following appendectomy in children has emerged from the success of fast-track surgery protocols, reducing hospital stays and building on outpatient surgery trends (54–56). However, there are concerns about racial and ethnic disparities in SDD rates (42, 43, 57, 58). Sullivan et al. conducted a retrospective study utilizing data from a US registry. Being Black or African American was linked to a lower likelihood of experiencing SDD, with an adjusted odds ratio of 0.85 [95% confidence interval (95% CI): 0.79–0.92; *p* < 0.0001]. In contrast, Hispanic ethnicity was associated with a higher likelihood of experiencing SDD, showing an adjusted odds ratio of 1.19 (95% CI: 1.12–1.25; *p* < 0.0001) compared to non-Hispanics (59). According to Oyetunji et al. who utilized data from a national US database in his study, children of African American and Hispanic descent had a lower likelihood of experiencing SDD than Caucasian and non-Hispanic patients (*p* < 0.05) (60). The severity of the illness, unrecognized patient social circumstances, and unequal hospital treatment could all contribute to these disparities (61). Addressing differences in race and ethnicity in SDD rates is crucial for equitable healthcare access. In addition to racial and ethnic disparities, studies from the US show that SDD had comparable or slightly better complication rates than admitted groups (*p* < 0.05), with Alkhoury, Halter, Gee, and Yangyang's studies highlighting these findings (62–65). To broaden our perspective and address healthcare resource allocation, it's equally essential to evaluate the cost-effectiveness of SDD. Zheng et al., in a systematic review and meta analysis, reported significantly lower hospital expenses for SDD patients (-$2,587) (66), Kashyap et al. found lower mean charges for SDD ($32,450 vs. $35,420) (67), and Yangyang et al. noted reduced appendectomy episode costs ($8,073 vs. $8,424) and high patient satisfaction (mean score: 9.4/10) (65). Gee et al. also report lower median costs for SDD ($29,150) compared to non-SDD ($34,827) (64). These findings support SDD's feasibility and cost-effectiveness, making it a crucial quality metric in pediatric surgery. Implementing SDD can reduce inpatient service utilization for this high-volume condition, possibly lower the incidence of hospital-acquired infections, and enhance patient and family satisfaction. Interventions such as cultural competence training for healthcare providers, enhanced communication strategies, community outreach, and policy interventions are essential steps toward reducing these disparities (66).

## 5 Conclusion

Appendicitis is the most common surgical condition in the pediatric ED, necessitating swift diagnosis and treatment to reduce morbidity (1). However, disparities in surgical care based on race and ethnicity contribute to poorer outcomes for pediatric patients (42).These disparities manifest in various aspects such as wait times, diagnostic accuracy, pain management, surgical access, hospital LOS, and SDD rates, as highlighted by this review article. Research consistently shows that minority populations experience worse outcomes, influenced by factors like distrust in the medical system, communication barriers, poor health literacy, and logistical challenges such as the inability to take a break from work or problems with transportation. Implicit bias within the healthcare system and discrepancies in pediatric subspecialty referral rates further exacerbate these inequities. Minority children are often treated by lower-volume providers and have less access to pediatric surgical subspecialists. Furthermore, ethnic and racial populations differ in their language competence, and a low level of English proficiency can make it more likely for medical professionals to miss appendicitis symptoms. In addition, the timing of presentations can vary according to socioeconomic status, race, and ethnicity; milder symptoms in early presentations may result in a delayed diagnosis. Interventions including improved communication techniques, cultural competency training, community engagement, and policy reforms for healthcare professionals are needed to address these gaps. Standardized procedures for diagnosis and treatment should be put into place, the staff should be more diverse, and ongoing professional development centered on healthcare fairness should be ensured. Healthcare systems can strive towards equal access to surgical care for all racial and ethnic groups by implementing these focused strategies, which will eventually improve patient care and results (43, 68). To lessen the gaps in surgical access for minority children, we hope that this work increases awareness among doctors and encourages improved surveillance, rapid-cycle quality improvement, and more research. One limitation of our review is that several of the cited articles are relatively dated, and incorporating more recent research would enhance the relevance and depth of the discussion.
